# Frozen vs. fresh cycles IVF outcomes: retrospective study from an Indonesian IVF centre

**DOI:** 10.1186/s13104-021-05585-w

**Published:** 2021-05-10

**Authors:** Wiryawan Permadi, Hartanto Bayuaji, Kevin Dominique Tjandraprawira, Dian Tjahyadi, Harris Harlianto, Yanni Melliandari Achmad, Nanang Winarto Astarto, Tono Djuwantono

**Affiliations:** 1 Department of Obstetrics and Gynaecology, Faculty of Medicine, Universitas Padjadjaran, Bandung, Indonesia; 2 Bandung Fertility Center, Limijati Women and Children Hospital, Bandung, Indonesia

**Keywords:** Embryo transfer, Frozen embryo transfer, Fertilization in vitro, Live birth

## Abstract

**Objective:**

To compare the live birth rates (LBR) and neonatal outcomes of frozen cycle in vitro* fertilization* (IVF) with fresh cycle IVF in the Indonesian population.

**Results:**

This was retrospective study using secondary data of IVF patients at a private fertility centre. Study recruitment was between 3/8/2018 and 31/12/2019. Total sampling included all patients undergoing oocyte retrieval and embryo transfer within recruitment period. Patients undergoing fresh IVF cycles and frozen IVF cycles were compared. 351 patients were recruited: 68.1% (239/351) underwent fresh cycles and 31.9% (112/351) frozen cycles. AMH was significantly higher in frozen cycle group (p = 0.04). Ovulatory disorder was significantly higher in frozen cycle group (p = 0.001). Among patients aged ≤ 30, fresh cycle group had significantly higher LBR (p = 0.02). Among those with ovulatory disorder, LBR was significantly higher with frozen cycle. No significant LBR difference was noted with other infertility causes. When stratified according to pregnancy order, frozen cycle patients had significantly higher birth lengths (p = 0.03) but not length of gestation nor neonatal birthweights. There was no significant difference in the proportion of biochemical pregnancy resulting in LBR (p = 0.08). To conclude, frozen cycle provided higher LBR among patients with ovulatory disorder but fresh cycle was beneficial among patients aged ≤ 30.

## Introduction

Assisted reproductive technology (ART) is available in Indonesia to help against infertility, including in vitro fertilization (IVF) and its associated more advanced techniques [1]. Regarding IVF, frozen embryo transfer (FET) has been deemed superior to fresh embryo transfer (ET) policy with regards to the primary outcomes, i.e. the live birth rate (LBR), and fewer complications, particularly ovarian hyperstimulation syndrome (OHSS) [2].

Frozen cycle has been advocated for distinct settings, such as for endometriosis-associated infertility, in which FET may improve implantation rates [3]. In another study, external maternal factors, such as body mass index (BMI), could modify IVF success among polycystic ovarian syndrome (PCOS) patients [4]. Currently, the debate persists and the lack of sufficiently robust evidence on LBR indicates that the superiority of the frozen strategy cannot be fully advocated yet.

There are so far limited data on the outcome of IVF cycles in Indonesia. Thus, this study aims to compare the LBR and the associated neonatal birthweights and birth lengths of frozen IVF cycles and fresh cycles.

## Main text

### Methodology

This was a retrospective study utilising secondary data from patient case notes as a clinical audit of IVF patients presenting to a private fertility center in Bandung, Indonesia. The study recruitment period ran between 3 August 2018 and 31 December 2019. We employed total sampling and included all patients undergoing oocyte retrieval and embryo transfer within the recruitment period. We excluded IVF patients having undergone oocyte retrieval before 3 August 2018 despite having their embryo transfer (ET) or frozen embryo transfer (FET) within the recruitment period.

Patient case notes and laboratory worksheets were retrieved to collect data on patient demographics and details on their procedures. Data on the embryo transfer were also collected, regarding cycle type, the number of embryos/blastocysts transferred and their grades. Embryo grading was done using the Gardner criteria. Patients were assigned to either cycle at the clinician’s discretion. Fresh cycle was defined as oocyte retrieval followed by cleavage-stage or blastocyst transfer in the same cycle. Frozen cycle was defined as oocyte retrieval followed by cryopreservation of all embryos (cleavage-stage embryos and/or blastocyst). Thawing was done in a subsequent cycle and the embryo(s) were transferred.

Beta-hCG levels were measured at 13/15 days post-embryo transfer and a beta-hCG level of 55 ng/mL established biochemical pregnancy [5]. For those achieving biochemical pregnancy, they were followed up by phone by the clinical staff for their pregnancy. Delivery details and their neonatal data were recorded. Patient participation was censored on 31 December 2019.

The ethical clearance for this study was granted by the Health Research Ethics Committee of Universitas Padjadjaran with the following Approval Number 1131/UN6.KEP/EC/2020. This study received no external funding.

### Statistical methods

Chi-squared test or Fisher’s exact test were performed on categorical variables, depending on their sample sizes. T-test or the Mann–Whitney U test was utilised when comparing numerical variables containing two groups of data. Significance was set at 0.05.

Data was entered into a customized database on Microsoft® Excel for Mac v16.16.3 (Microsoft®, Redmond, USA). They were analysed using Statistical Product and Software Solutions (SPSS) for Mac v.23 (IBM Corp, Armonk, New York, USA).

## Results

Between 3 August 2018 and 31 December 2019, there were 351 patients divided into 2 groups: 239 patients (68.1%) undergoing fresh cycle IVF and 112 patients undergoing frozen cycle (31.9%). Table 1 presents the demographics of our patients. There was not a significant difference in age among those undergoing fresh cycle vs. frozen cycle. Using Mann–Whitney U Test, patients undergoing fresh cycles had significantly lower AMH levels than those undergoing frozen cycles.

**Table 1:** Patient Demographics

Variables	Fresh cycle (239)	Frozen cycle (112)	p-value
Age (median, interquartile range)^a^	33 (29–37)	33 (30–37)	0.8
Abdominal surgery history^b^			
Yes	127 (53.1%)	52 (46.4%)	0.3
No	111 (46.9%)	60 (53.6%)	
AMH level (median, interquartile range)^a^	2.02 (1.3–3.28)	2.54 (1.32–4.7)	0.04
Types of infertility^b^			
Primary infertility	179 (74.9%)	79 (70.5%)	0.4
Secondary infertility	60 (25.1%)	33 (29.5%)	
Ovulatory disorder^b^	51 (21.3%)	44 (39.3%)	0.001
Tubal factor infertility^b^	97 (40.6%)	37 (33.0%)	0.2
Uterine factor infertility^b^	61 (25.5%)	38 (33.9%)	0.1
Endometriosis^b^	37 (15.5%)	16 (14.3%)	0.8
Male-factor infertility^b^	183 (76.6%)	87 (77.7%)	0.8
Protocol^b^			
Long-protocol	163 (68.2%)	65 (58%)	0.06
Short-protocol	76 (31.8%)	47 (42%)	
Oocytes retrieved (median, range)^a^	9 (5–14)	10 (6–17)	0.05
Mature oocytes (median, range)^a^	8 (4–12)	9 (5–16)	0.03
Embryos number (median, range)^a^	3 (2–5)	4 (3–7)	0.005
Excellent embryos count (median, range)^a^	2 (1–3.5)	2 (1–4)	0.8
Good embryos count (median, range)^a^	1(0–2)	2 (1–3)	0.006
Blastocysts number (median, range)^a^	3 (3–5)	4 (3–6)	0.1
Excellent blastocysts count (median, range)^a^	2 (1–3)	2 (1–4)	0.8
Good blastocysts count (median, range)^a^	1(0–2)	1 (0–2)	0.04
Biochemical pregnancy^b^			
Yes	71(29.7%)	44 (39.3%)	0.04
No	168 (70.3%)	62 (60.7%)	
Live birth^b^			
Yes	57 (23.9%)	16 (14.3%)	
No	182 (76.1%)	96 (85.7%)	0.05

^a^Mann-Whitney U test was used for continuous variables

^b^Fisher’s exact test was used for categorical variables

The frozen cycle group had significantly more patients with ovulatory disorder than the fresh cycle group (39.3% (44/112) vs. 21.3% (51/239), p-value 0.001) but not tubal factor infertility, uterine factor infertility, endometriosis and male-factor infertility.

Whilst both groups did not differ significantly in the number of oocytes retrieved, the median number of mature oocytes of the frozen group was significantly higher (9 (5–16) then the fresh cycle group (8 (4–12), p-value 0.03.

The frozen group had significantly more embryos (4 (3–7) vs. 3 (2–5), p = 0.005). Whilst the 2 groups had similar number of excellent embryos, the frozen group still had significantly more good embryos (2 (1–3) vs. 1 (0–2), p-value 0.006).

Of those patients whose embryos were cultured into blastocysts, the 2 groups didn’t differ significantly in the number of blastocysts produced, except that the frozen group had fewer good embryos than the fresh cycle group (p = 0.04).

There was not a significant difference in the biochemical pregnancy rate of both groups (p = 0.7) but the LBR was significantly higher in the fresh-cycle group than the frozen group (p = 0.05).

In Table 2, we found that among patients aged 30 years and less, the fresh cycle group had significantly higher LBR than the frozen group (p = 0.02). We found a statistically significantly higher LBR among those with ovulatory disorder treated with frozen cycle (p = 0.04).

**Table 2:** Live birth rate between groups

Variables	Live birth rates among		p = value
	Fresh cycle	Frozen cycle	
Live birth rate ≤ 30 year olds	27 (47.4%)	2 (12.5%)	
Live birth rate > 30	30 (52.6%)	14 (87.5%)	0.02
Ovulatory disorder^a^			
Present	10 (17.5%)	7 (43.8%)	
Absent	47 (82.5%)	9 (56.2%)	0.04
Tubal factor infertility^a^			
Present	27 (46.6%)	4 (25%)	0.2
Absent	30 (53.4%)	12 (75%)	
Uterine factor infertility^a^			
Present	14 (24.6%)	5 (31.3%)	0.8
Absent	43 (75.4%)	11 (68.7%)	
Endometriosis^a^			
Present	9 (15.8%)	2 (12.5%)	1
Absent	48 (84.2%)	14 (87.5%)	
Male-factor infertility^a^			
Present	47 (82.5%)	11 (68.8%)	0.3
Absent	10 (17.5%)	5 (31.2%)	

^a^Fisher’s Exact test was used on the above categorical variables

We assessed the neonatal outcomes of both groups (Table 3). Among singleton pregnancies, the birth length was statistically significantly higher among frozen cycle pregnancies (p = 0.03) but not the length of gestation nor the birth weight (p = 0.08; 0.06). Among multiple pregnancies, none of the neonatal outcomes were significantly different between the two groups.

**Table 3:** Neonatal outcomes among singleton and multiple pregnancies

Neonates from singleton pregnancies	Fresh cycle	Frozen cycle	p-value
Length of gestation^a^	244.5 (219–263)	244 (200–260)	0.8
Birth weight^a^	2800 (2200–3900)	3200 (3009–3654)	0.06
Birth length^a^	48 (44.5–52)	49 (45–51)	0.03

Neonates from Multiple Pregnancies	Fresh cycle	Frozen cycle	p-value
Length of gestation^a^	231 (224–235)	228 (222–232)	0.6
Birth weight^a^	2150 (1835–2500)	2545 (2222–2572.5)	0.97
Birth length^a^	45 (44–47)	47 (45.5–47)	0.97

^a^ Mann–Whitney U Test was used for all above continuous variables

We found no significant LBR difference, regardless of whether the biochemical pregnancy came from a fresh ET or FET (p = 0.09). Only in fresh ET would the presence of excellent embryos significantly increase LBR(p = 0.02). The association was not seen for excellent embryos towards live birth rate in FET (p = 0.4). We also failed to observe the same association between excellent blastocysts and the LBRs (p = 0.06).

## Discussion

We found statistically significantly higher LBR among the fresh-cycle patients. Whilst our frozen cycle group is considerably smaller than the other group, our result agrees with Stormlund et al.’s study [6]. They found that among regularly menstruating women, frozen strategy did not improve the LBR when compared to fresh cycle strategy [6]. Their findings warrant caution towards the use of liberal frozen cycle strategy in the absence of strong indications such as ovarian hyperstimulation syndrome (OHSS) and preimplantation genetic testing (PGT) [6].

Our study found that among patients with ovulatory disorder, there was a significantly different proportion in LBR between the fresh cycle group (10/47, 17.5%) and the frozen group (7/16, 43.8%) (p = 0.04). However, there was no significant difference in the biochemical pregnancy rate of the 2 groups (p = 0.2). This is similar to Chen et al.’s study [7]. They found that among patients with polycystic ovarian syndrome (PCOS), FET resulted in significantly higher number of live births despite no significant difference in biochemical pregnancy rate [7]. This is a promising finding, as this would suggest that for those with PCOS, FET should be advocated to produce higher LBR. Furthermore, the relationship shown by Chen et al. was replicated in our study, despite our smaller number of subjects.

We found that among patients with endometriosis, there was no significantly different proportion in the biochemical pregnancy and LBR between the groups (p > 0.999; p = 0.361 respectively). Results have been conflicting. Mohamed et al. and Bourdon et al. reported that deferred embryo transfer through frozen strategy was significantly associated with higher cumulative pregnancy rate [3, 8]. However, Feichtinger et al. reported the opposite [9]. Again, the lack of difference in our study might have been due to the lack of power as the number of endometriosis patients in our dataset was < 50.

We discovered that there were roughly similar live birth rates among mothers ≤ 30 years old and > 30 years old in the fresh cycle group. However, in the frozen cycle group, mothers aged > 30 years old significantly delivered more neonates than those ≤ 30 years old. This is different from a very large-scale study in China by Zhu et al. [10]. Another study by Wang et al., though, could possibly corroborate our results [11]. They found that among women whose progesterone concentration > 1 ng/mL, increasing age was associated with significantly higher LBR with freeze-only transfer cycles [11]. Whilst progesterone was not measured among our patients, the rising progesterone might have been the reason behind the very high success rates for frozen cycles among those > 30 years old [11].

The lack of association between presence of excellent embryos and live birth rates in FET was surprising, despite the presence of such association among ET cycles. Whilst our lack of association is surprising, it might have been due to the relatively few pregnancies from FET patients over 1.5 years of data recruitment in our study. Another reason might have been due to suboptimal endometrium among our patients. With more patients opting for the frozen cycle at our facility, more data would be generated to allow for a repeat and more powerful analysis in the future.

The frozen cycle was associated with higher median birthweight (3200 (3009–3654) than the fresh cycle neonates (2800 (2200–3900)). Despite this association not being statistically significant, the trend was present and we suspect that the lack of significance was due to the fewer subjects of the frozen group when compared to the fresh cycle group. This was seen in the overall birthweight comparison, as the median birthweight of the freeze-cycle neonates (whilst not adjusting for the order of pregnancies) was statistically significantly higher than the fresh cycle neonates (p = 0.01). This may have been due to the good quality of the cryopreserved embryos [12]. Furthermore, the lower birthweight among fresh ETs might have been caused by the higher likelihood of abnormal placentation due to the over-estrogenized uterine environment [12].

Our study’s strengths include the following. First, it is the first for our centre and to our knowledge, the first in our country to publish IVF data and compare the outcomes of fresh cycle IVFs against frozen-cycle IVFs. Second, our centre is among the top centres with high IVF cycles per year in Indonesia and our patients originate not just from the neighbouring cities but also from distant provinces.

To conclude, we did not find any significant differences in both the biochemical pregnancy rate and the LBR between frozen cycles and fresh cycles. We also failed to observe significant differences in the LBRs when stratified according to the aetiology of infertility except by their ages.

## Limitations

However, our study has a number of limitations. Being the first in our country to compare the outcome of the 2 cycles, there are no data yet to compare our results with. Furthermore, our study is small, compared to the large-scale studies and randomized controlled trials (RCTs) that have been published internationally and hence, the lack of power. Third, our study is single-centred thus the presence of selection bias and attrition bias cannot be ignored.

Furthermore, we didn’t analyse the effect of the endometrial preparation on the success rate of FET pregnancies. With the limited dataset that we have, stratifying FET pregnancies according to their endometrial preparations would further reduce the power of the analysis. A bigger dataset would be necessary for a meaningful analysis.

We did not have data on potential confounding variables, such as patient BMI and smoking status and disabling adjustment for such confounding variables.

## Data Availability

The datasets generated during this study are not available due to patient confidentiality reasons but are available upon reasonable written request to the corresponding author.
